# Economic valuation of informal care provided to people after a myocardial infarction in France

**DOI:** 10.1186/s12913-019-4637-5

**Published:** 2019-10-28

**Authors:** Hugo Rabier, Hassan Serrier, Anne-Marie Schott, Nathan Mewton, Michel Ovize, Norbert Nighoghossian, Antoine Duclos, Cyrille Colin

**Affiliations:** 1 Hospices Civils de Lyon, Pôle de Santé Publique, Service d’Evaluation Economique en Santé, 162, avenue Lacassagne – Bâtiment A, 69424, Cedex 03 Lyon, France; 2 Université de Lyon, Université Claude Bernard Lyon , Université Saint-Etienne, HESPER EA 7425 F-69008 Lyon, F-42023 Saint-Etienne, Lyon, France; 3 Hospices Civils de Lyon, Cellule Innovation, Délégation à la Recherche Clinique et à l’Innovation, Lyon, France; 4 Hospices Civils de Lyon, Groupement Hospitalier Est, Centre d’Investigation Clinique, INSERM 1407, Bron, France; 5 Department of Neurology, Hospices Civils de Lyon, Université Lyon 1, Lyon, France; 6 Department of Stroke Medicine, Hospices Civils de Lyon, Université Lyon 1, Lyon, France

**Keywords:** Informal care, Myocardial infarction, Contingent valuation method, Willingness to pay, Cost, France

## Abstract

**Background:**

The aim of this study was to estimate the mean cost per caregiver of informal care during the first year after myocardial infarction event in France.

**Methods:**

We used the *Handicap-Santé* French survey carried out in 2008 to obtain data about MI survivors and their caregivers. After obtaining the total number of informal care hours provided by caregiver during the first year after MI event, we estimated the value of informal care using the proxy good method and the contingent valuation method.

**Results:**

For MI people receiving informal care, an annual mean cost was estimated at €12,404 (SD = 13,012) with the proxy good method and €12,798 (SD = 13,425) with the contingent valuation method per caregiver during the first year after myocardial infarction event.

**Conclusions:**

The present study suggests that informal care should be included more widely in economic evaluations in order not to underestimate the cost of diseases which induce disability.

## Background

Outcomes of health economic evaluations are one of the criteria used by decision-makers for market access of medical devices and new drugs. Economic evaluations are required for all new drugs in Australia, Hungary, New Zealand and Sweden; upon request one year after launch in Germany; and for specific drugs and devices in England and Wales [1]. In France, the use of health economic evaluations in the decision-making process is more recent. Since 2013, a health economic evaluation is requested by the French national evaluation agency named *Haute Autorité de Santé* (HAS) only for an innovative and expensive drug or medical device [2]. From the HAS perspective, innovative products are defined as those for which the manufacturers claim a moderate to major improvement of the clinical benefit compared to that provided by existing treatments. Expensive products refers to a product which have a significant impact on the statutory national health insurance expenditures or an impact on the organization of care, professional practices or patient care conditions. In parallel, the HAS published a methodological guide for the economic evaluation [3] in which it is stipulated that if the viewpoint adopted for the evaluation was societal, informal care cost should be included. Informal care represents the care provided by family members, close relatives, friends, or neighbors who are not a professional caregiver and not trained to provide care, and without monetary compensation [4].

Costs of informal care can represent a significant proportion of the total cost induced by the disease, especially for chronic diseases. Even if several valuation methods of informal care exist, as the proxy good (PG) method, the opportunity cost method and the contingent valuation (CV) method, these costs are not always taken into account in the economic evaluations of chronic diseases and more rarely for acute illness like myocardial infarction (MI). Many international and European studies report that informal care represents a major economic impact and a real burden for caregivers associated with chronic diseases like dementia or cancers [5–9]. This is not really a surprising finding for chronic diseases, but may be less suspected for acute illness such as acute coronary syndrome although there are long-term effects in part due to chronic complications (namely congestive heart failure). Acute coronary syndrome refers to a spectrum of clinical presentations ranging from those for ST-segment elevation MI to presentations found in non–ST-segment elevation MI or in unstable angina [10]. In France, informal care valuation studies are mainly focused on Alzheimer’s Diseases and disabled elderly people [11–14].

MI represents the main cause of mortality among coronary heart disease patients in Europe [15, 16], and in France, the incidence of MI is estimated to be around 120,000 cases every year [17]. MI can cause difficulties in performing realize activities of daily living (ADLs) and instrumental activities of daily living (IADLs), that may involve the need of an informal caregiver [18]. However, no evaluation of the cost of informal care in MI has ever been conducted. The absence of informal care cost in economic evaluations can be explained by the fact that this is not a chronic disease but can also be explained by the small amount of data available or studies carried out from a societal perspective. Moreover, inclusion of informal care in economic evaluation induces methodological issues because caregivers can meet difficulties to quantify hours of informal care provided. When caregiver and care receiver live together, the caregiver can overestimate the informal care duration if he fails to distinguish additional housework provided to assisted person and “normal” daily home activities [19].

In spite of the recommendations from the methodological guide for the economic evaluation [3] to include cost of informal care in medico-economic evaluation, few studies provided these data and only concern chronic diseases. We propose to estimate the informal care costs for acute illness taking the case of MI as an example.

The main objective of the present study was to estimate the mean cost per caregiver of informal care during the first year after an MI event in France because MI is associated with significant increases in functional disability at short-term [18] and the majority of second events occur in the first year following the MI event [20]. To do this, we used the CV and the PG methods based on data provided by the *Handicap-Santé* survey [21, 22]. We also realized a systematic review to estimate an approximate overall mean cost of MI management during the first year in order to quantify the relative weight of informal care.

## Methods

### Data source

Data was collected from the prospective 2008 Health and disabilities households survey (*Enquête Handicap-Santé*), carried out in the French general population by the French National Institute of Statistics and Economic Studies and the Department of Research, Studies, Evaluation and Statistics. The first part of this survey, named *Handicap-Santé*, *volet Ménages* (HSM) [21], was conducted in ordinary households and collected socio-demographic, economic and health information from approximately 30,000 participants. It examined the consequences of health problems on functional limitations, care-seeking (professional and informal), professional and daily activities, and degree of dependence. Degree of dependency was determined in the survey according to the Katz Index of Independence in ADLs (bathing, dressing, go and using the toilet, getting in and out of bed and enter then leave his seat, incontinence and feeding) [23]. This instrument is used to assess functional status as a measurement of ability to perform activities of daily living activities. People were scored for dependence in each of six activities. A score of 6 indicates that individual was able to make the 6 ADLs, and below six an impairment to realize at least one ADL. In the present study, care receivers with a score equal to 6 were classified as “autonomous to realize the 6 ADLs” and below 6, individuals were classified in the “not able to realize at least one ADL” category. The second part of the survey, *Handicap-Santé, volet Aidants informels* (HSA) [22], was based on telephone or face-to-face interviews with approximately 5000 informal caregivers of the people interviewed in the first part of the survey. Information was collected about personal characteristics, professional activity, social environment, description of care provided (type of activity and number of hours per week) and impact of provided care on family, professional and social environment.

We selected in the HSM survey, people survivors of MI less than 12 months before the interview and who received informal care. Then, we linked care-receivers with their caregiver who answered to the HSA survey.

#### Economic valuation

We estimated the economic value of informal care in two steps. First, we used the number of informal care hours provided per week and per caregiver stated in the HSA survey. Caregivers may encounter difficulties distinguishing informal care from daily “normal” home activities when they live with the assisted person and can overestimate the duration of informal care. To avoid an overestimation, we computed two estimates of the value of informal care: one in which the stated duration of informal care was restricted to 10 h (“restricted scenario”), and the other without restriction (“unrestricted scenario”). We chose a maximum of 10 h because 90.6% of caregivers stated an informal care duration inferior or equal to 10 h per day.

Then, we valued care time using the PG and the CV methods. The PG is a revealed preference method which values informal care time at the labour price of a market substitute by applying the market wage rate of a close substitute. Assuming that informal and formal care are perfect substitutes, the wage rate of a professional caregiver may be used to value informal hours [19]. However, care hours should be valued according to the task carried out, care at the market wage of a home-care nurse and housework tasks at the market wage of housekeeper. Care hours can also be valued using the French legal minimum gross hourly wage [13] set at €9,76 on 1 January 2017 [24]. We valued care hours using the minimum hourly wage because the HSA survey data cannot determine precisely the number of hours according to the type of care performed by caregivers. We assumed that the minimum hourly wage is near to home-care nurse and housekeeper mean wage rate because the qualification level of person working in the home personal services care sector is low [25].

The CV is used to obtain the monetary value per hour of informal care from the perspective of the informal caregiver on the basis of stated preferences. The caregiver assesses the maximum amount of money willing to pay (WTP) to be replaced for one hour of informal care. This method allows us to capture all relevant aspects of informal care due to its sensitivity to the different situations informal caregivers faced with, and it reflects their preferences [12]. The latter was collected in the HSA survey through the following open question regarding their WTP to be substituted for one additional hour of caregiving:

*‘Suppose you have the opportunity to be replaced for one hour of care in the week. What is the maximum amount would you be willing to pay for this hour of care? Mind that this amount corresponds to a reduction of your budget’.*

If caregivers met difficulties to answer, a second question proposing a fixed payment scheme:

*‘To help you, I am going to show you a table with different values. You could start to remove all amounts of which you are sure do not pay. Then, select all amounts whose you are sure to pay. Lastly, select the maximum amount would you be willing to pay’*.

Some caregivers may find it unethical to indicate a monetary value and it may lead to protest answer illustrated by a willingness to pay stated equal to “0” or caregivers could refuse to give a WTP value to the first question. WTP value equals to zero was considered as “protest answer” only when caregiver gave a justification. We noted the number of “protest answers” but we integrated WTP value equal to “0” in the analysis.

Informal care cost estimates were presented in 2017 euros. WTP values stated were inflated to 2017 euros using the appropriate Consumer Price Index published by the French National Institute of Statistics and Economic Studies [26].

## Results

### Study sample

**Fig. 1:**
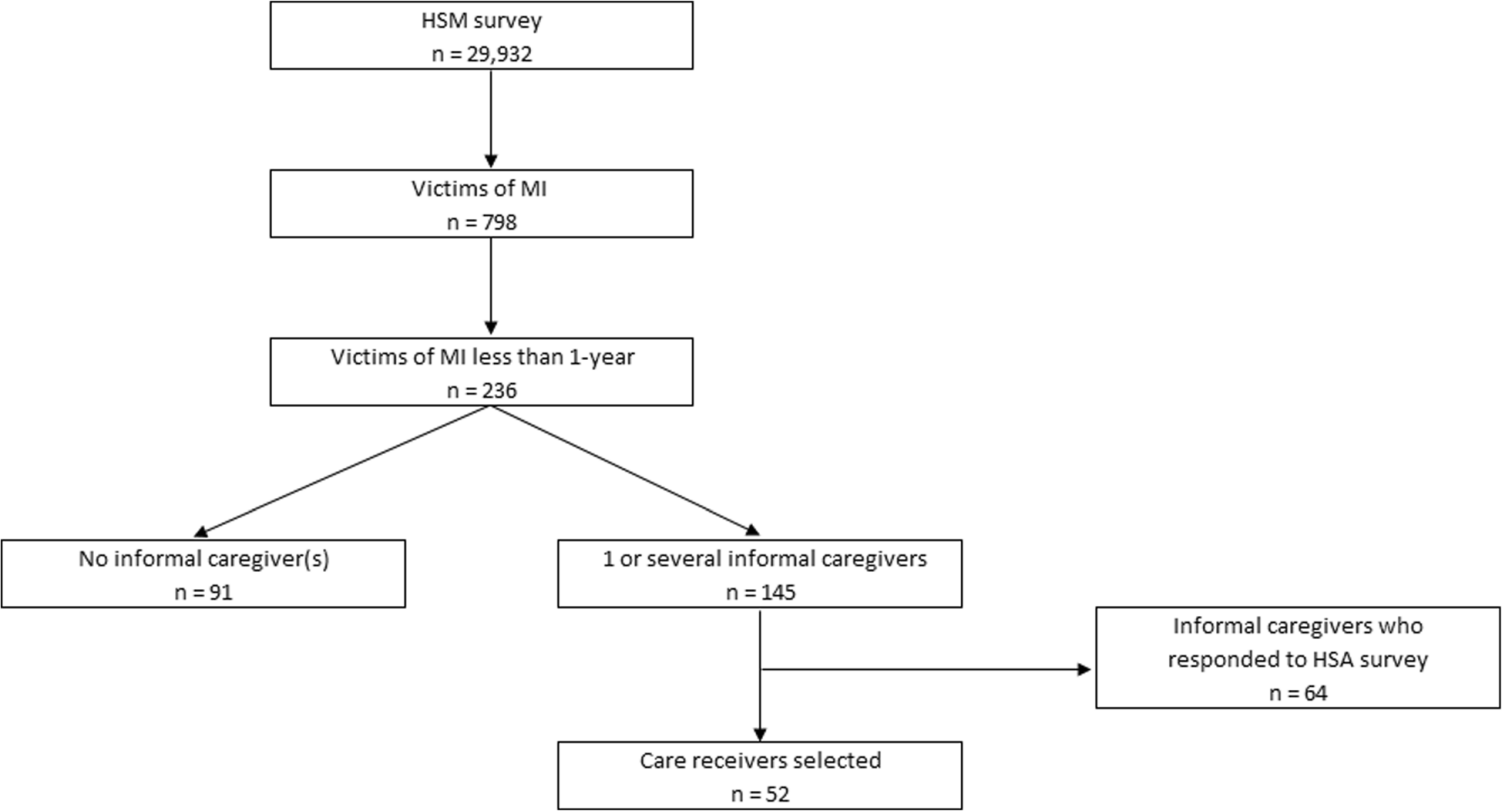
Analytical sample from *Handicap-Santé* survey (2008)

We selected 798 people survivors of MI in HSM survey. Among this selection, the sample was restricted to 236 survivors of MI less than 12 months before the survey. Then, we retained individuals who received care by one or several informal caregivers (*n* = 145). Among these individuals, we selected MI survivors for whom caregivers who responded to the HSA survey (*n* = 64). Our final sample was 52 care receivers and their 64 caregivers (Fig. 1).

Table 1 shows characteristics of care receivers and their caregivers. Average age of care receivers is 71.0 years old and the distribution of genders is balanced (women 48.1%). Forty four percent of care receivers are single and 70.3% are « autonomous » to perform activities of daily living according to the Katz index. Care receivers autonomous to realize the 6 ADLs received mainly care as “management organization/medical appointment scheduling” (80.7%), “medication management” (75.0%) and “moral support” (60.5%). Whereas care receivers with an impairment to realize at least one ADL received care as “toileting” (49.1%), “bathing” (47.2%), “dressing” (60.0%) and “moral support” (75.3%). Caregivers are mainly partner (50.0%) and son or daughter (37.5%), and more than half lives with their care recipient (73.1%).

**Table 1:** Characteristics of care receivers and their caregivers, care receivers who refused to respond

Characteristics (%)	Informal caregivers (*n* = 64)	Care receivers (*n* = 52)	Care receivers with caregivers who refused to respond (*n* = 93)
Age (mean)	54.9	71.0	72.4
Female	71.9	48.1	37.6
Married/couple	62.9	55.8	54.2
Relationship with care receiver			
Partner	50.0	–	48.7
Brother/sister	6.2	–	5.1
Son/daughter	37.5	–	40.0
Other family member	3.1	–	3.6
Friend	1.7	–	2.6
Cohabitation with care receiver	73.1	–	–
Educational level			
No degree/primary school	48.4	73.1	76.0
Secondary school	40.6	23.1	21.7
University degree	6.2	3.8	2.3
Refusal	4.8	0.0	0.0
Dependency			
Autonomous	–	70.3	72.3
Dependent	–	29.7	27.7
Duration of care provided per day (hours)	3.5	–	–
Autonomous	2.9	–	–
Dependent	4.9	–	–
WTP stated (euros)	10.7	–	–
Autonomous	9.6	–	–
Dependent	11.2	–	–

### Economic value of informal care

The average WTP stated by caregivers to be replaced for one hour of care is €10.7 (SD = 6.83; median = 10; IQR [5–15]; range [0–25]). 8 caregivers stated a WTP value equal to “0”. Among these null WTP, 3 caregivers expressed a disagreement (“it is my duty” or said “I want to do it”.) and are considered as “protest answers” (Table 1).

The average annual value of informal care per caregiver obtained using the CV method is €12,798 (no restriction scenario) for a daily average duration of informal care of 3.5 h (Table 2). The average value of informal care for autonomous people is substantially less than dependent people: respectively €10,228 versus €20,270 for the no restriction scenario and €10,086 versus €17,169 for the restriction scenario. A sensitivity analysis with the “protest zeros” data was carried out. The “protest zeros” values were replaced by the mean WTP of similar caregivers according to the Index Katz score of their care receivers, if they lived together and if they had a work activity. An average annual value of informal care per caregiver at €14,062 (no restriction scenario) and €13,061 (restriction scenario) was obtained.

**Table 2:** Average annual value of informal care per caregiver according to valuation method and dependency (2017 euros)

	Autonomous (SD)	Dependent (SD)	Total (SD)
*Contingent Valuation Method*			
No restriction	10,228 (10,253)	20,270 (12,984)	12,798 (13,425)^a^
Restriction	10,086 (9872)	17,169 (12,171)	11,888 (11,848)^b^
*Proxy Good Method*			
No restriction	10,377 (10,402)	21,099 (12,604)	12,404 (13,012)
Restriction	10,233 (10,016)	17,776 (11,4604)	11,522 (10,643)

No restriction scenario: exact durations of care stated by caregivers. The mean is 3.5 h of informal care/day/caregiver

Restriction scenario: durations superior to 10 h were censored to a maximum of 10 h per day. The mean is 3.2 h of informal care/day/caregiver

^a^Total (SD) including zero protest = €11,921 (13,528)

The average annual value of informal care per caregiver obtained using the PG method is €12,404 (no restriction scenario). The average value of informal care for autonomous people is substantially less than dependent people: respectively €10,337 versus €21,099 for the no restriction scenario and €10,233 versus €17,776 for the restriction scenario.

## Discussion

Among MI survivors receiving care provided by informal caregiver, we estimated a mean cost per caregiver of informal care at €12,798 using the CV method and €12,404 using the PG method. The findings further suggest that informal care cost cannot be considered negligible in MI and underline the importance of incorporating informal care costs in economic evaluations to assist policy-makers in the allocation of limited resources for healthcare provision.

A study estimated the informal care cost for patients with Alzheimer’s disease at €11,269 per year for a mild Alzheimer’s Disease severity and €28,883 for a moderate to severely Alzheimer Disease severity with the PG method in France [27]; with the opportunity cost method, cost of informal care was estimated to be €15,706 and €39,864 per year, respectively. Another study valued informal care associated with dementia in Central Europe and obtained a mean duration of informal care reported to be 31.23 h per week corresponding to an average annual cost of informal care estimated at €21,126 [8]. Our lower cost estimation of informal care is not surprising in comparison with a chronic disease, but according to the same study, the average annual cost of informal care associated with stroke was estimated at €6576 with a mean duration of 23.98 h per week. It can be explained because the latter estimated a mean of informal care with several study results taking into account stroke events diagnosed more than one year. Whereas we only focused on during the first year after MI, the majority of second event occurring in the first year [20]. In consequence, MI survivors required more care in the first year than long-term. These comparisons shows our results are consistent with the literature and that informal care cost could vary according to the valuation method used, the level of dependence, but also country studied [8].

To value informal care cost, we used the PG and CV methods. Although the two valuation methods were based on different shadow prices, overall cost estimations did not vary significantly. Despite the fact that the CV method captures the preference heterogeneity of caregivers, several studies showed that caregivers have difficulty giving a WTP value on a hypothetical market. Hence, caregivers tented to refer to the value of one hour on the professional home care market [28] which could explain these similar estimations. Another valuation method exists: the opportunity cost method This method estimates the cost of informal care by using the market wage of the caregiver or of a home care service only depending on the work and leisure time forgone to provide informal care and does not take into account the heterogeneity preferences of people [29]. Caregiver preferences are heterogeneous because they are affected by individual characteristics, caregivers do not reason only by duration of care provided and can give more importance to the type of care activities, the home distance with the care receivers or the frequency of care provided. The CV method captures heterogeneity preferences of caregivers via the WTP and follows the welfare theory based on individual preferences [30]. Moreover, to compare informal care cost in MI patients with other countries, the CV method could be used to give a representation of the informal care to be taken into account the specificities as the health systems and cultural aspects with the WTP. But, it is necessary to compare results of other studies with caution because results may have varied depending on the diseases studied, the level of dependency, the current policies and cultural differences between the countries concerned. For instance, in the Nordic countries and the Netherlands, aid policies for care receivers to employ a professional home care or a housekeeper is more widespread than in France, Germany, or England. The existence of a professional care can have an impact on the WTP stated by the caregiver [31]. Another alternative is to use the willingness to accept (WTA) in the CV method to capture caregivers’ preferences. Several studies shown that WTA values for non-market goods are two to five times higher than the WTP values [32–34]. WTA is known to suffer from a bias due to the lack of budget constraint and to loss aversion [35]. Lastly, the National Oceanic and Atmospheric Administration (NOAA) Panel Report recommends the use of WTP in contingent valuation studies because the report claims that “respondents are more likely to exaggerate the compensation they would require than their willingness to pay [36].

Some limitations of the present study should be noted. The CV method contains a hypothetical bias because this method is only focused on monetary value. Caregivers may find it unethical to indicate a monetary value and it may lead to protest answer illustrated by a willingness to pay stated equal to “0” [14]. To consider protest answers, we might also have included an estimation without the WTP answer equal to zero when it is specified in comment that the caregiver do not want to pay to be substituted. But we choose the conservative assumption to avoid overestimation and carried out a sensitivity analysis substituting the “protest zeros” by the mean WTP of similar caregivers according to sociodemographic characteristics. On the other hand, as caregivers were not confronted with a real market; informal caregivers can have difficulties estimating a monetary value on the hypothetical market which could result in an under or over-estimation of the WTP.

The estimation of informal care is based on a restricted sample of 64 caregivers and important information about informal care is loss because 81 caregivers did not want to respond to the HSA survey. Care receivers linked with the caregivers who did not want to respond received 2.11 h per day on average. The *Handicap-Santé* survey dates back to 2008 and can represent a limitation because management (greater use of reperfusion therapy and recommended medications) and secondary treatments of MI have evolved over the last 10 years, resulting in significant improvements in outcomes [37]. These outcome improvements induce a modification of formal and informal care needs However, the *Handicap-Santé* survey is the sole database providing informal care data about MI survivors and individual estimations of the WTP for informal caregivers of MI survivors that allow us to use the CV method. The CV method is less frequently used because of the use of survey which are costly, complex and time-consuming.

Health economic evaluations are useful for decision makers particularly for pricing negotiation and reimbursement of health product. Krol et al. [38] recalculated Incremental cost-effectiveness ratios (ICERs) from studies including informal care and they showed that the inclusion of informal care may have an impact on cost-effectiveness outcomes and may even exceed medical costs. In a study which analysed how the inclusion of social costs could change ICERs of economic evaluations of interventions for Alzheimer’s disease. Among 55 economic evaluations identified in which both health care payer and societal perspectives were included, authors showed that in 3 of them, the new intervention became cost-effective when social costs were taken into account [39]. However, despite guidelines, informal care costs are not routinely considered in health economic evaluations.

Results suggest that informal care can represent a non-negligible economic impact and should be included in economic evaluations not only for chronic diseases but also for acute illnesses in order not to underestimate the cost of diseases, which induce dependencies. Including informal care in economic evaluations allows reporting to the French decision-makers the role of caregivers, promoting their social recognition with the aim of considering an appropriate mixture of care in order to improve caregiver quality of life via introduction of measures, for instance, more flexibility to adapt working-time and respite home programmes.

## Conclusions

Taken together, the present study provides a quantitative indication on the fact that informal care represents a significant economic impact for informal caregivers.

## Data Availability

The datasets used and/or analysed during the current study are available from the corresponding author on reasonable request.

## Abbreviations

ADL: Activities of Daily Living
CV: Contingent Valuation
HAS: *Haute Autorité de Santé*
HSA: *Handicap-Santé Aidants*
HSM: *Handicap-Santé Ménages*
IADL: Instrumental Activities of Daily Living
ICER: Incremental Cost Effectiveness Ratio
MeSH: Medical Subject Headings
MI: Myocardial Infarction
NOAA: National Oceanic and Atmospheric Administration
PG: Proxy Good
PRISMA: Preferred Reporting Items for Systematic Reviews and Meta-Analyses
SD: Standard Deviation
WTA: Willingness To Accept
WTP: Willingness To Pay
